# Profiles of subjective health among people living alone: a latent class analysis

**DOI:** 10.1186/s12889-021-11396-2

**Published:** 2021-07-07

**Authors:** Tytti P. Pasanen, Nina Tamminen, Tuija Martelin, Katariina Mankinen, Pia Solin

**Affiliations:** 1 Finnish Institute for Health and Welfare, Equality / Mental health, P.O. Box 30, FI-00271 Helsinki, Finland; 2 Finnish Institute for Health and Welfare, Knowledge Management and Co-creation / Knowledge Base for Health and Welfare Management, Tampere, Finland; 3 Finnish Institute for Health and Welfare, Equality / Non-Discrimination and Gender Equality, Helsinki, Finland

**Keywords:** Single occupancy households, Health status, Psychological well-being, Social life, Quality of life

## Abstract

**Background:**

Living alone has increased globally and especially in Finland where 45% of all households are single occupancy. Epidemiological research has found that living alone a risk factor for a wide range of adversities related to quality of life but the rapidly-changing demographics of people living alone calls for a more detailed investigation of their subjective health status.

**Methods:**

Using a cross-sectional survey sent for a random sample of Finnish residents in single-person households (*n* = 884), we explored with latent class analysis whether the respondents form different health profiles based on the three health dimensions defined by the World Health Organization: physical, social, and mental well-being. The identified groups were then compared in terms of demographic characteristics with the χ^2^ test and quality of life using linear regression models. Sensitivity analyses were run using more refined, manual 3-step BCH method.

**Results:**

Four distinct health profiles were found: Languishing (4%), Managing (35%), Healthy (30%), and Flourishing (31%). The groups differed in most socio-demographic aspects such as marital and employment status, but not in terms of geographic location or gender (apart from group Languishing that contained more men). Controlling for these socio-demographic differences, all groups showed different average levels of perceived quality of life to the expected direction.

**Conclusions:**

Our findings suggest that people living alone are indeed a very heterogeneous group in terms of subjective health. Instead of seeing living alone as a mere risk for low quality of life, concept of living alone should be understood more broadly both in public discussion and scientific research.

**Supplementary Information:**

The online version contains supplementary material available at 10.1186/s12889-021-11396-2.

## Background

Living alone has become more and more common around the world, with 35% of all households in EU-27 and 31% in the OECD-32 comprising of one adult [1, 2]. This trend has been especially strong in Finland where currently 45% of all households are one-person households [3]. Moreover, the share of single occupancy households has increased in all adult age groups in the past 10 years [1]. Thus, the group of people living alone has become increasingly diverse. Research on those living alone has, nevertheless, been largely focused on late adulthood [4].

Living alone is known to be associated with a number of adversities such as poor mental and physical health, loneliness and mortality (for example, [5–8]). These issues have been thought to derive from a lack of social support and companionship that a partner usually provides, and greater financial strain including higher living costs [9]. Accordingly, living by oneself – often based on marital status – has been generally considered a risk factor in epidemiological research that needs to be ‘controlled for’ by treating it as a comparison group to those who are married or cohabiting with a partner. This categorisation ignores the diversity of people living alone in their various life situations and health statuses [10].

The concept of health, as defined by World Health Organisation (WHO) more than 70 years ago, is a “complete state of physical, social, and mental well-being” [11]. However, seeing these dimensions as dichotomies, that is, one either qualifies as ‘healthy’ in these aspects, or not, has been criticised [12–14]. Instead, more recent views suggest that the different dimensions that together form the experience of health, are continuums, and none of them should be considered solely by a presence or an absence of a diagnosed illness [12, 13]. The physical dimension, therefore, also refers to the capability and resilience to cope under physiological stress [14], and mental well-being to positive emotional and psychological well-being [15]. The social dimension of the WHO health definition has been questioned the most [12, 13] and, for example, some views see it as a part of mental well-being [15]. Nevertheless, social well-being encompasses multiple aspects of social behaviours and their perceived quality (e.g. [16–18]). Although the physical, social, and mental aspects of health are conceptually different, they have been found to correlate at least moderately in population samples and it is possible to experience deterioration in one dimension while having a high level in another [12, 19, 20].

A related concept to subjective health is quality of life. WHO defines quality of life as “individual’s perception of their position in life within the context of the culture and value systems in which they live and in relation to their goals, expectations, standards and concerns” [21]. Therefore, quality of life is a broader concept than health and well-being [22]. Rather than being a dimension of health, quality of life refers to how the different dimension of health affect an individual’s quality of life [21]. This effect is always subjective but it is influenced by one’s cultural, social and environmental context [21]. As a consequence, subjective health status is inevitably one of the factors contributing to the sense of quality of life [22]. This view has been supported empirically, with quality of life correlating strongly with mental health and moderately with social relationships and general health [23, 24].

With the regard to health and well-being of people living alone, a major gap in the literature is that studies have mainly been conducted in the field of gerontology with focus on the elderly [4, 25]. When the older age groups are assessed, those living alone cluster in same groups (for example, [26–28]), which hinders assessing variation within them. Tentative evidence, however, suggests that the health status of people living alone in comparison to those not living alone varies by age: in mid-adulthood (35–64 years), those living alone have a poorer health but at older ages, their health is better compared with those not living alone [4]. Our study is one of the first to target people living alone in all ages of adulthood, thus it provides more comprehensive information on their health and well-being. With their rapidly increasing share, the health of people living alone across all life stages is a crucial public health matter.

This study focuses on uncovering the subjective health status of people living alone, with three broader objectives. First one is to explore whether people living alone form distinct groups that differ in their profiles of health, assessed by the physical, mental, and social dimensions following the WHO definition of health [11]. Although the methodology used to form these groups, latent class analysis (LCA), is an exploratory statistical tool, we are in particular interested in groups that might experience varying levels of subjective health in different dimensions. Second, if distinct subjective health profiles are identified, we assess the socio-demographic and geographic profiles of these groups to better understand their backgrounds. Third, we examine if and how much these groups differ in their perceived quality of life, with the aim of understanding how closely subjective health and quality of life are associated among people living alone.

## Methods

The study followed the principles of the Declarations of Helsinki. All methods were performed in accordance with the STROBE checklist for observational studies and the relevant parts of the SAMPL guidelines.

### Data

A survey questionnaire, as part of the project “Positive mental health, quality of life and social support experienced by people living alone in Finland”, was sent by mail to a random sample (using a simple random draw) of 3000 Finnish-speaking residents of Finland aged 18 or over who were registered as living in a one-person household, in the autumn of 2019. The official register only allows one address per resident and hence, the sample may have included people whose under-aged children live with them part-time or who were married with a partner registered in another address (due to, for example, being institutionalised or working abroad). After one reminder, 911 completed questionnaires (either electronic or postal) were returned, of which we excluded 27 due to the recipient declaring living with someone most of the time (*n* = 17), duplicate responses (*n* = 5), another person replying on behalf of the recipient (*n* = 3), or inappropriate response style (*n* = 2). After this, the final sample was *n* = 884 (response rate 28%). Female gender and older age groups were overrepresented in comparison to their shares in the Finnish sub-population of adult people living alone (based on data from the end of 2018 [3]). Hence, the data was weighted based on gender and age (in 5-year intervals) by calculating adjustment weights for each respondent so that the weighed dataset had equal gender x age distributions to the sub-population of one-person households in Finland. For example, in older women (who were overrepresented), the weight was less than 1, while younger men (underrepresented) had a weight greater than 1.

### Measures

#### Health

Mental well-being dimension of health was measured with the Warwick-Edinburgh Mental Well-Being Scale (WEMWBS), a measure of positive mental health validated in different languages and cultures [29, 30]. The scale consists of 14 positively-phrased items regarding the past 2 weeks (for example, “I’ve been feeling confident” and “I’ve had energy to spare”), rated on a scale from 1 ‘None of the time’ to 5 ‘All of the time’. The total score, with a range of 14–70, is the sum of all items [31]. The scale has been translated into Finnish. The age-adjusted mean in recent Finnish population study (for aged > 30 years) was 52.5 points for both men and women [32]. In our weighed sample, the mean was 49.4 (Table 1; see Additional file 1 for age-stratified means).

**Table 1:** Distributions of the study variables

Variable	Range or category	*n*	Proportion / mean (SD)
Positive mental health (WEMWBS)	14–70	840	49.4 (9.7)
Perceived general health		876	
	Good/rather good		58.7%
	Average/rather poor/poor		41.3%
Social provisions (SPS)	24–96	798	77.6 (12.2)
Quality of life (EUROHIS)	1–5	869	3.8 (0.7)
Gender		882	
	Male		46.8%
	Female		53.2%
Age in years		884	
	18–29		19.4%
	30–64		44.2%
	65-		36.4%
Marital status		872	
	Single		50.9%
	Divorced		26.6%
	Widowed		18.4%
	Married/cohabiting		4.1%
In a relationship		864	
	No		76.5%
	Yes		23.5%
Education		876	
	Primary		21.2%
	Secondary		37.5%
	Tertiary		41.3%
Employment status		870	
	Employed/studying		50%
	Unemployed		13.1%
	Retired/other		36.9%
Region (NUTS2)		884	
	Helsinki-Uusimaa		26.6%
	South Finland		25.6%
	West Finland		24%
	East & North Finland		23.8%
Urbanicity		870	
	City/town centre		30.3%
	City/town suburb		49.9%
	Population centre in a rural area		13.4%
	Sparsely populated rural area		6.4%

Physical dimension of health was asked as a single item ‘In general, would you say your health is’, rated on a scale from 1 ‘Good’ to 5 ‘Poor’. A single question has been found a valid measure of physical aspects of health [33], with good predictive power on morbidity and mortality [34, 35]. In the Finnish population (aged > 30 years), 62% of men and 63% of women perceive their health as good or rather good [36], while this share was 59% in our sample (Table 1).

Social dimension of health was assessed with the Social Provision Scale (SPS), comprising of 24 items on six different dimensions of social relationships: integration (for example, ‘There are people who enjoy the same social activities I do’), attachment (for example, ‘I feel a strong emotional bond with at least one other person’), reliable alliance (for example, ‘There are people I can depend on to help me if I really need it’), reassurance of worth (for example, ‘I do not think other people respect my skills and abilities’), guidance (for example, ‘There is someone I could talk to about important decisions in my life’), and nurturance (for example, ‘No one needs me to care for them’), developed by Cutrona & Russell [18] based on Weiss’s theory on social provisions [37]. Each dimension comprises of four items, two positively and two negatively-phrased, rated on a scale from 1 ‘Completely disagree’ to 4 ‘Completely agree’. The sum of all items (negative ones reverse-scored), ranging from 24 to 96, forms the final score. The structure of the dimensions has been inconsistent but the total score has shown good internal consistency [18, 38].

#### Quality of life

Quality of life was measured with the 8-item EUROHIS-QOL questionnaire [22]. The items measure satisfaction with one’s physical and psychological well-being, social relationships and living environment. Each item is rated on a scale from 1 to 5, and their mean comprises the final score. The age-adjusted mean in the Finnish population (of aged > 30) is 4.0 [39] and in our weighed sample, the mean was 3.8 (Table 1).

#### Socio-demographic and geographic factors

*Gender* was examined with two categories, female or male, due to too few responses in the ‘other’ category (*n* < 5). *Age* was categorised into three groups (18–29, 30–64, and 65-) based on previous studies stating different levels of well-being in early, middle and late adulthood [4]. *Marital status* comprised of four categories: single, divorced, widowed, and married/cohabiting. *Relationship status* was assessed with the question ‘Are you in a steady relationship?’ with the options ‘yes’ and ‘no’.

Highest obtained *education* was categorized into three levels: comprehensive (primary or comprehensive school), upper secondary (higher secondary school or vocational school), and higher (degree obtained in university, applied university, or [former] vocational college). *Employment status* was likewise grouped into three categories; employed/studying (including part-time employment), unemployed (including disability pension, temporarily laid-off, or similar), and retired/other.

*Region* was examined with the NUTS 2 categorisation that divides the mainland into four larger areas: Helsinki-Uusimaa and South, West, and East & North Finland, based on information on the respondents lower-level region obtained from the registry. *Urbanicity* was based on the question ‘In what type of neighbourhood do you live in?’, with the options ‘City/town centre’, ‘City/town suburb’, ‘Population centre in a rural area’ and ‘Sparsely populated rural area’.

### Statistical analyses

#### Latent class analysis

The aim of latent class analysis (LCA) is to classify similar observations into latent, that is, unobserved, groups based on their responses to selected indicators [40]. The LCA analyses were conducted using mixture modeling option in Mplus version 8.5 [41] which can weight cases and handle missing values and observed variables that are binary, ordered categorical, or continuous. The classes were formed using the MLR estimator, based on full information maximum likelihood estimation robust to non-normal observed variables, meaning that all respondents with at least one valid response (*n* = 882) were included in the formation of the latent classes. SPS and WEMWBS were standardised and specified as continuous observed variables. Perceived general health was specified both ordinal and binary (cut-off at good/rather good and average/rather poor/poor). Within-class variances and covariances were allowed to vary across groups. Our initial aim was to run each specification for 1–7 latent classes. In case the optimal solution was not replicated at least twice, the number of random starts was iteratively doubled up until 16,000 random starting values, after which we discontinued the analysis.

As there is no agreement of a single indicator to decide the optimal number of groups, nor clear cut-off values for evaluating the goodness-of-fit of a solution, these evaluations were conducted by jointly considering statistical model fit, adequate class sizes, and interpretability [40]. Statistical fit was determined by the three available information criteria (IC, with smaller values indicating better fit): Aikake’s (AIC), Bayesian (BIC), and sample-adjusted BIC; entropy (with higher values indicating better separation); and the Vuong-Lo-Mendell-Rubin Likelihood Ratio Test (VLMR-LRT) test that compares the current solution to the solution with one less class [40, 42]. The recommended bootstrapped LRT [43] was not available with the weighed data. It is not uncommon that different fit indices point to different solutions as superior, and in such a case we closely examined and compared the soundness of the solutions content-wise, taking into consideration that class-specific sample sizes should be adequate to assess them against other variables (described in the next two sections [40]).

In the rest of this article, we refer to the classes found in the LCA analysis as ‘groups’.

#### Group comparisons

The demographic, socio-economic and geographic characteristics (defined in “Socio-demographic and geographic factors” section) of the groups were assessed descriptively and their proportions compared with the χ^2^ test. If the χ^2^ test indicated differences in the overall distributions (using the criteria of *p* < .05), groupwise proportions were compared. These comparisons were conducted with SPSS version 26. The proportion of missing data were below the rule-of-thumb of 5% that can be considered negligible [44] (Table 1; 0–18, that is, 0–2%, missing responses per covariate), and hence missing values were excluded pairwise in each comparison.

#### Connection with quality of life

Linear regression models were conducted to assess the groups in terms of quality of life. Three models with different predictors were tested: first containing only the latent groups (unadjusted model), second with the covariates (“Socio-demographic and geographic factors” section; covariate-only model), and third with latent groups and the covariates (adjusted model). Missing values were excluded listwise. In addition to the regression coefficients, the models were evaluated with the variance explained in the outcome and residual distributions. These analyses were conducted in R version 3.6.0 [45].

#### Sensitivity analyses

First, because two items of EUROHIS-QOL conceptually overlapped with the health indicators (satisfaction with one’s health and social relationships), we ran a sensitivity model excluding these items from the quality of life measure.

Second, we re-examined the associations between the latent groups, socio-demographic factors, and quality of life taking into account observation-specific probabilities of group membership, using the manual 3-step BCH method, developed by Bolck, Croon, and Hagernaars [46]. Although the estimates, taking into account uneven classification probabilities, are more accurate with this method compared with the traditional ‘classify-analyse’ approach applied in this paper, it is not uncommon to encounter issues such as negative variances due to small-sized groups [47]. For this reason, we had to exclude region from the analysis and combine some categories of the socio-demographic covariates for the analysis to converge, meaning that the results are conceptually close but not exactly comparable to the main reported analysis. This BCH analysis was conducted using Mplus version 8.5 [41], applying the syntaxes provided in [47].

Third, to consider the varying relationship between age and subjective health [4], we re-ran the latent group analysis stratified by the age groupings (18–29, 30–64, and ≥ 65 years) using the ‘knownclass’ option in Mplus (syntaxes adapted from [48]). However, because 1) our sample sizes in each stratified analyses were below or at the minimum of the recommended 300–1000 cases for latent class analysis [40] and 2) these stratified analyses failed reach a solution with optimal statistical fit and clear interpretation, we have concerns about the validity of these analyses and, hence, these results are not reported.

## Results

### Best-fitting solution

The information criteria were consistently smaller in solutions where general health was specified binary versus ordinal. For example, in the solutions for 2–5 groups, the sample-adjusted BIC varied between 6342 and 6480 with the ordinal and 5096–5209 with the binary specifications. Content-wise the groups were similar in both specifications. With the better-fitting binary specification, the optimal number of groups was four (Table 2). VLMR and LMR rejected most models with more groups in favour of less groups (which in not uncommon [42]), but the fit improved in terms of other criteria as the number of groups increased. Although in the 5-group solution the information criteria was smaller (in terms of BIC, only trivially) and entropy was slightly greater (.77 vs .73), the characteristics of the groups were largely the same. The additional fifth group constituted a small number of individuals (*n* = 20) that were a part of the fourth group in the 4-group solution.

**Table 2:** Model fit information for 1–5 groups with general health specified as binary and variances free to vary across groups. In bold: the chosen optimal solution (based on fit and content)

Number of groups	Log-likelihood	Free parameters	AIC	BIC	Adjusted BIC	Entropy	VLMR-LRT	Class counts
1	− 2714.6	6	5441.2	5469.9	5450.9			882
2	− 2581.3	13	5188.5	5250.7	5209.4	0.65	0.00	563, 319
3	− 2540.9	20	5121.7	5217.4	5153.9	0.54	0.63	235, 365, 282
**4**	**− 2512.5**	**27**	**5079.1**	**5208.2**	**5122.4**	**0.73**	**0.12**	**266, 306, 35, 274**
5	− 2486.8	34	5041.6	5204.1	5096.2	0.77	0.27	34, 308, 272, 247, 20

### Latent class descriptions

**Fig. 1:**
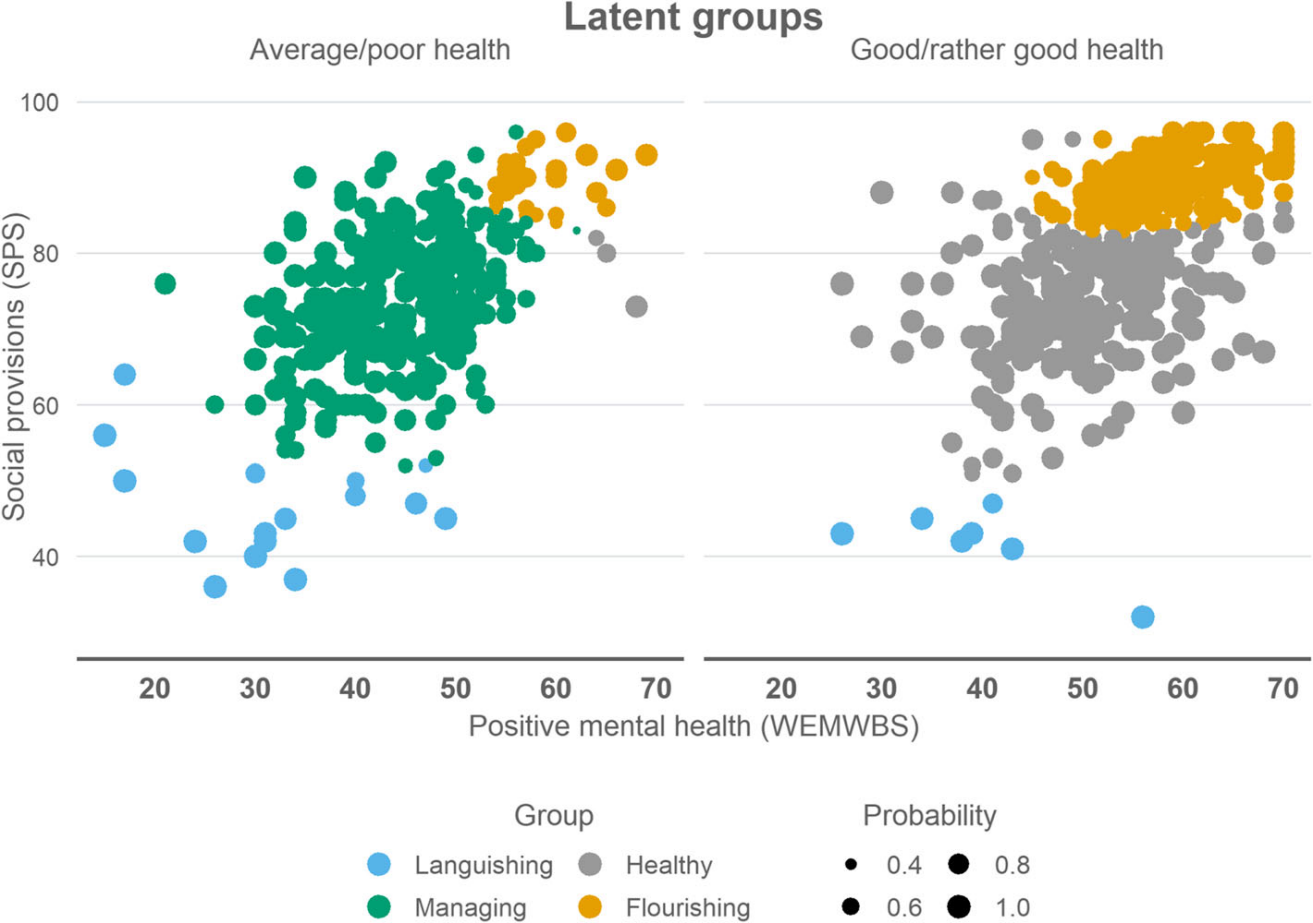
Scatterplots showing the distributions of the latent groups (*n* = 882) and probabilities to belong to the assigned group

The first group (*n* = 35, 4.0% of the sample) was by far the smallest, with extreme values in the lower ends in at least one of the health dimensions. Mean levels of both WEMWBS (30.9, sd = 10.2) and SPS (44.4, sd = 7.5) were low. Most (74.3%) perceived their general health as average or poor; those whose health was good/rather good had extremely low values on SPS (Fig. 1). We labelled this group “Languishing”.

All members in the second group (*n* = 307, 34.7%) perceived their health as average or poor. The mean level of WEMWBS was below the sample mean (43.8, sd = 6.7). In SPS, there was a lot of variation but the overall level was near sample mean (74.1, sd = 9.0). This group was named “Managing”.

In the third group (*n* = 266, 30.2%), almost all (98.9%) respondents perceived their health as good or rather good (Fig. 1). Both WEMWBS (mean = 49.3, sd =8.1) and SPS (mean = 74.0, sd =8.1) varied around average levels in the sample. This group was labelled “Healthy”.

In the fourth group (*n* = 274, 31.1%), most members (89.3%) perceived their health as good or rather good; those who did not, had high scores on both WEMWBS and SPS. Nearly all showed high levels of SPS (mean = 89.3, sd = 3.2), whereas WEMWBS varied slightly more around average and high levels (mean = 57.6, sd = 5.5). This group was named “Flourishing”.

### Socio-demographic and geographic characteristics of the groups

**Fig. 2:**
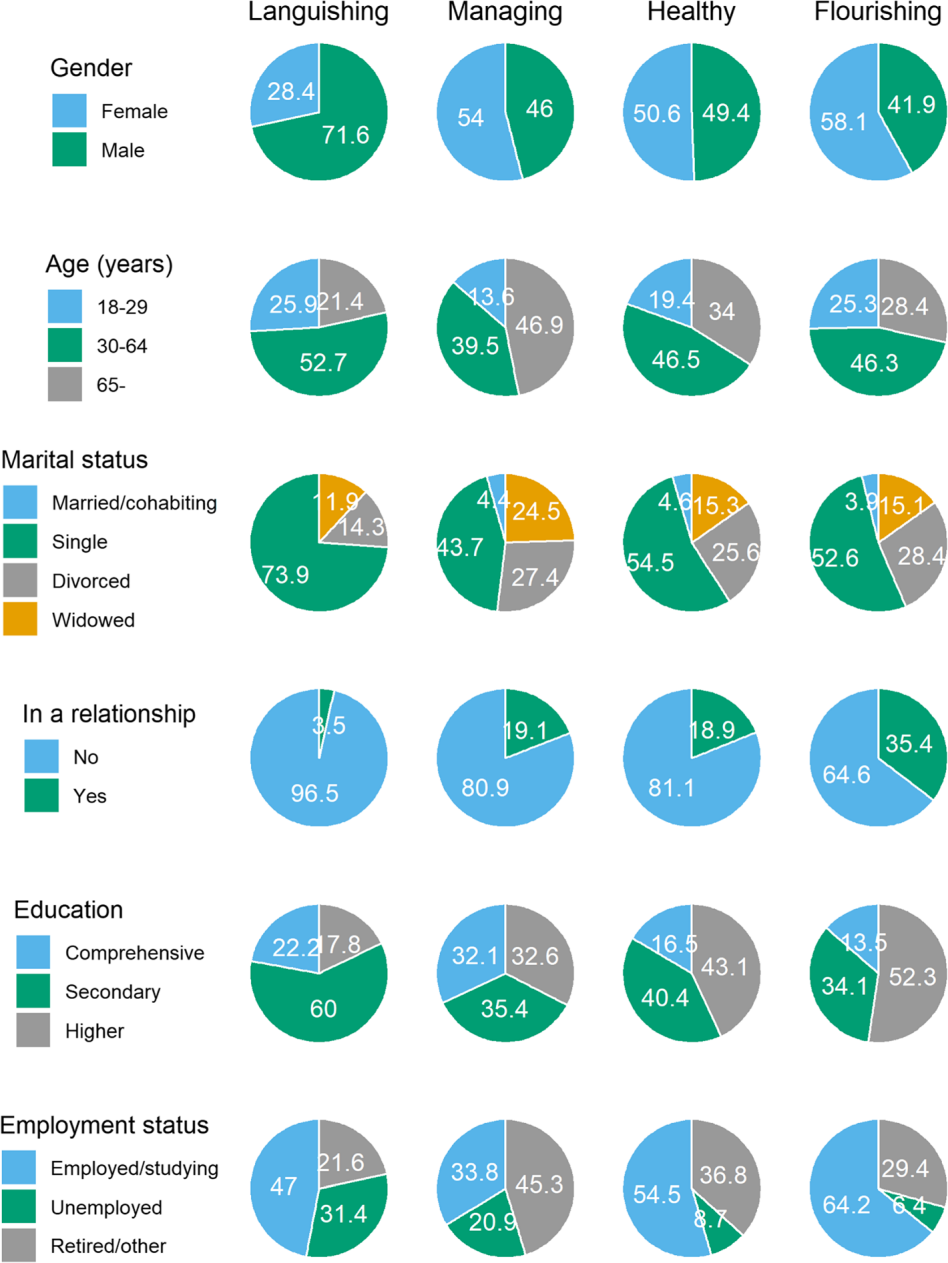
Socio-demographic distributions of the latent groups

The *gender* distributions were equal in groups Healthy, Managing and Flourishing (Fig. 2; see Additional file 2 for the 95% confidence intervals of these shares). Group Languishing contained more men (72%) than the other groups (χ^2^ = 11.1, df = 3, *p* = .011). In terms of *age* (χ^2^ = 29.4, df = 6, *p* < .001), the share of 30–64-year-olds was equal in all groups (40–53%). Groups Languishing and Flourishing contained slightly more young adults (25–26%) than Managing (14%), while the share of over 65-year-olds was greater in group Managing (47%) than in the other groups (21–34%).

Being single by *marital status* (χ^2^ = 21.8, df = 9, *p* = .010) was the most common category in all groups, and it was the most common in group Languishing (74%), followed by Healthy (55%) and Flourishing (53%). Widows were more common in group Managing (25%) than in groups Healthy (15%) and Flourishing (15%). The share of divorced was approximately equal in all groups (14–28%). Only a small share was identified as married/cohabiting (0–5%). Substantially greater proportions were, however, *in a relationship* (χ^2^ = 36.1, df = 3, *p* < .001): this was the most common for the members of group Flourishing (35%), equally common in groups Healthy and Managing (19%) but rare in group Languishing (4%).

Regarding *education* (χ^2^ = 52.8, df = 6, *p* < .001), having only completed comprehensive school was more common in group Managing (33%) than in groups Healthy and Flourishing (14–17%). In group Languishing, having completed secondary education was more common (60%) than in other groups (34–40%). Group Flourishing was the most highly-educated, with 52% having completed higher education, while this share was 33–43% in groups Healthy and Managing, and only 18% in group Languishing.

All groups differed in terms of their *employment status* (χ^2^ = 76.2, df = 6, *p* < .001). Working/studying was the most common in group Flourishing (64%), followed by groups Healthy (55%) and Languishing (47%), and the least common in group Managing (34%). In groups Languishing and Managing, the share of unemployed was greater (31 and 21%, respectively) than in groups Healthy and Flourishing (9 and 6%, respectively). In group Managing, being retired (45%) was more common than in the other groups (22–37%).

The distributions of *urbanicity* (χ^2^ = 6.731, df = 9, *p* = .665) and geographical *region* (χ^2^ = 9.216, df = 9, *p* = .418) were equal in all groups.

### Predicting quality of life

Mere group membership explained 43% of the variance in quality of life, and inclusion of socio-demographic covariates increased this only a little (*R*^2^ = .48) (Table 3). In the covariate-only model R^2^ was .15 (results presented in Additional file 3). Residuals were approximately normally distributed in the adjusted model, although slightly negatively skewed, suggesting more predicted values were overestimated than underestimated. No heteroscedasticity or non-linearity was, however, apparent.

**Table 3:** Linear regression models predicting quality of life (EUROHIS-QOL8), *n* = 809. Estimates in bold: *p* < .05. CI = compatibility interval

		Unadjusted (*R*^2^ = .43)			Adjusted (*R*^2^ = .48)		
Explanatory variable	(Category)	b	95% CI	*p*	b	95% CI	*p*
Latent group (ref. Healthy)	Languishing	**−1.26**	[−1.68; −0.85]	<.001	**− 1.13**	[− 1.51; − 0.75]	<.001
	Managing	**− 0.59**	[− 0.69; − 0.48]	<.001	**− 0.56**	[− 0.66; − 0.45]	<.001
	Flourishing	**0.42**	[0.32; 0.52]	<.001	**0.39**	[0.29; 0.49]	<.001
Gender (male vs female)					−0.02	[− 0.11; 0.08]	.744
Age group (ref. < 30 years)	30–64 years				−0.02	[− 0.18; 0.15]	.836
	> 65 years				0.15	[−0.08; 0.37]	.203
Marital status (ref. Single)	Married/cohabiting				−0.03	[−0.23; 0.17]	.776
	Divorced/separated				−0.01	[− 0.12; 0.09]	.800
	Widowed				**0.13**	[0.01; 0.26]	.032
Employment status (ref. employed/studying)	Unemployed				**−0.33**	[− 0.5; − 0.16]	<.001
	Retired/other				−0.09	[− 0.25; 0.08]	.298
In a relationship (vs not)					0.08	[−0.05; 0.21]	.205
Education level (ref. Primary)	Secondary				−0.02	[− 0.14; 0.1]	.717
	Tertiary				0.07	[− 0.03; 0.18]	.175
Region (NUTS2; ref. South Finland)	Helsinki-Uusimaa				0.02	[−0.1; 0.14]	.730
	West Finland				0.03	[−0.08; 0.14]	.617
	East & North Finland				0.06	[−0.07; 0.19]	.378
Urbanicity (ref. City/town centre)	City/town suburb				0.04	[−0.06; 0.14]	.426
	Population centre in a rural area				−0.08	[− 0.21; 0.05]	.220
	Sparsely populated rural area				−0.13	[−0.28; 0.03]	.102

In the adjusted model, compared with the group Healthy, groups Managing and Languishing had significantly lower quality of life (by .56 and 1.13 points, respectively) and the group Flourishing had .39 points greater quality of life score, controlling for the socio-demographic and geographic characteristics (Table 3). These estimates were similar in both models.

In addition, those who were unemployed had lower quality of life compared with respondents who were employed or studying (b = −.33, *p* < .001), and widowed had higher quality of life compared with those who were single by their marital status (b = .13, *p* = .032) when group membership was included in the model. Other socio-demographic or geographic variables were not associated with quality of life in the adjusted model.

### Sensitivity analyses

In the first sensitivity model, with satisfaction with one’s health and relationships excluded from the quality of life measure, the results were content-wise largely the same and the variance explained was only two percentage points lower. In the estimates, the greatest difference was in the coefficient of the group Managing (b = −.47, 95% CI = [−.58, −.36], *p* < .001). These results suggest that only a small share of the strong association between the latent groups and quality of life is due to the two conceptually overlapping items.

In the second sensitivity analysis using the more refined BCH 3-step approach that weighs cases based on group membership probabilities, the results were, likewise, largely the same. The covariate distributions within the latent groups were no more than four percentage points different to the unweighted shares reported in “Socio-demographic and geographic characteristics of the groups” section (Additional file 5). In the regression model, too small cell proportions in group Languishing caused issues in estimation, and we had to combine some categories of marital status and urbanicity, and exclude region, for the model to converge (see Additional file 4 for detailed results).

## Discussion

This study has shown that people living alone vary substantially in their subjective health, assessed in physical, social, and mental dimensions. Our objectives were to identify the groups, compare their socio-demographic and regional characteristics, and examine if they exhibit different levels of quality of life. Accordingly, we identified four latent groups that showed different profiles of health. These groups were, in turn, strongly connected to perceived quality of life, in line with prior studies [23, 24]. Comparing the group profiles to those found in other samples is complex because no similar study, to our knowledge, has looked at the whole population of adults living alone. Ng et al. [49] also examined physical, social and mental dimensions of health (albeit with different indicators) among the elderly population in Singapore, and found two distinct groups: those with average and those with lower level of health in all dimensions. These groups are similar to the groups Healthy and Managing in our study.

From the presumption that health is consisted of three dimensions that are conceptually separate but empirically related [12, 19, 20], our latent groups showed that it is not rare to experience largely varying levels of health in different dimensions. For example, while physical and mental well-being were, on average, perceived greater in group Healthy compared with Managing, their average evaluations on social well-being were equal. Similarly, in group Flourishing, some (albeit a minority) with very high levels of mental and social well-being rated their physical health as average or poor. These findings suggest that experiencing a low level of physical (or mental) health does not necessitate deteriorated social provisions.

All groups had different socio-demographic profiles. Group Healthy represents the ‘average’ in terms of both health and socio-demographic characteristics; they did not stand out from the others in terms of any particular characteristics. By contrast, the demographic profile of group Managing largely differed from the others: they were older, less educated, and more commonly widowed and retired or unemployed. Low education and unemployment have been consistently shown to be risk factors for low general and mental health in earlier studies [6, 50], while being retired and/or widowed are more likely indicative of older age than risk factors per se [4]. However, the group that showed the lowest levels of well-being, Languishing, was characterised by different socio-demographic factors. This group was dominated by men who were mainly single (in both marital and relationship status), unemployed, in mid-adulthood, and with an upper secondary degree than most other groups. In contrast, on the positive end of the well-being dimensions, was group Flourishing. This group shared known socio-economic characteristics that have explained high levels of health and well-being in past research, including higher education level, being employed or studying, and being in a relationship [49].

Importantly from the public health perspective, in two groups (Healthy and Flourishing), consisting of nearly two thirds of our sample, the average levels of subjective health and quality of life were similar to the rest of the Finnish population [39]. Furthermore, group Flourishing, with very high level of health and wellbeing, comprised nearly a third of our sample, whereas having extremely low levels on one or more health dimensions (group Languishing) was much rarer. This emphasises the fact that while the results from previous epidemiological studies consistently show that living alone is a risk factor for low levels of health [5–7], their variation in subjective health is considerable.

Although the well-being groups differed in their socio-economic profiles, none of the socio-demographic factors defined the groups. For example, all groups contained some persons who were unemployed, and even if group Languishing predominantly consisted of males, more than a quarter of them were female. As expected, those who were married (or cohabiting) were rare in our sample. This suggests that married couples living apart (due to e.g. institutionalisation or relocation for work) are a marginal phenomenon, although we have no official statistics to confirm this. The evidence for the beneficial effects of having a significant other tends to highlight marriage as a pathway to greater well-being and quality of life [51]. In people living alone, being in a steady relationship could provide similar advantages [52]. In our analyses, being in a relationship was most common in the group showing highest levels of well-being and quality of life (Flourishing) although majority (65%) were single. As all members in this group rated their social provisions high, it seems that having a ‘significant other’ is not always necessary for having one’s social needs fulfilled. The kind of social relationships –in terms of both quantity and quality– that enable high levels of social well-being in people living alone deserves more detailed investigation.

Finland, similar to the rest of the world, is becoming more urbanised [53], and single occupancy households tend to cluster in urban centres [3]. Nevertheless, urbanicity was not connected to the health profiles identified in this study, suggesting that so far the health status of people living alone has not diverged between rural and urban dwellers. Likewise, the groups were geographically equally represented across Finland. This is somewhat contrary to existing evidence that have shown regional differences in perceived health status [54] and mental health [55], though these refer to the entire Finnish population and not solely on people living alone. The regional variation in the proportion of inhabitants living alone (e.g. [56]) may even be one of the factors contributing to these regional health differences.

This study had its limitations. First, all health measures were self-reported evaluations and thus, subjective to social desirability and memory bias. Second, while the survey response rate was moderate, 28%, and the sample was weighed in terms of age and gender, it is possible that some subgroups of people living alone were underrepresented. For instance, earlier studies have suggested that in health surveys, non-participants tend to have poorer health status than participants, which could apply to our study (e.g. [57, 58]). Third, this was the first study to look at the whole population of people living alone in a single country. Replications are needed to verify the health profiles in Finland and to assess whether similar groupings apply to other countries. It is also possible that the found health profiles can be found in the whole population, not just among those living alone, which is a matter for prospective studies to assess. Fourth and finally, our sample was not large enough to assess whether and how the subjective health profiles differ across different age (and life) stages. We strongly encourage future investigations with larger population samples to conduct such an analysis to better understand the complexity and variety of the interplay between living alone and subjective health across age.

In summary, people living alone are a diverse group in terms of physical, mental, and social dimensions of health as well as quality of life, and many of them show similar levels of health to the general population in Finland. The strongest socio-demographic correlates of health in people living alone are consistent with those covering the whole populations: employment status, education, and relationship status [51, 52]. Although a small share of people living alone had extremely low levels in at least one health dimension, having higher levels was more common. These results highlight the need to broaden the understanding of the circumstances and health associations among people living alone, in both public discussion and scientific research, as the number of people living alone increases in the society.

## Supplementary Information

**Additional file 1.** Sample descriptives (weighed sample), stratified by age group.

**Additional file 2.** Socio-demographic distributions and the 95% CIs of the latent groups.

**Additional file 3.** Covariate-only model explaining quality of life (EUROHIS-QOL8).

**Additional file 4.** BCH-corrected LCA model with covariates and quality of life (EUROHIS-QOL8) as distal outcome.

**Additional file 5.** Socio-demographic distributions of the latent groups, weighed by classification probabilities using the BCH method.

## Data Availability

The data is stored at the Finnish Institute for Health and Welfare (THL), and it is available for research purposes upon reasonable request from Dr. Pia Solin.
