# Supplementary material for: Profiles of subjective health among people living alone: a latent class analysis
Source: BMC Public Health. 2021 Jul 7;21:1335. doi: 10.1186/s12889-021-11396-2 (PMC8261976; doi:10.1186/s12889-021-11396-2)
Supplement: Supplementary file 1 — Additional file 1. Sample descriptives (weighed sample), stratified by age group. [file 12889_2021_11396_MOESM1_ESM.docx]

**Additional file 1.** Sample descriptives (weighed sample), stratified by age group.

|  |  | Proportion / mean (sd) | | |
| --- | --- | --- | --- | --- |
| Variable | Category | 18-29-year-olds  (n=172) | 30-64-year-olds (n=391) | >65-year-olds (n=321) |
| Positive mental health (WEMWBS) (n=840) | | 49.2 (10.4) | 48.3 (10) | 50.9 (8.9) |
| Perceived general health (n=876) | |  |  |  |
|  | Good/rather good | 70.8 % | 62.1 % | 47.9 % |
|  | Average/rather poor/poor | 29.2 % | 37.9 % | 52.1 % |
| Social provisions (SPS) (n=798) | | 80.1 (11.9) | 77.4 (13.1) | 76.3 (10.8) |
| Quality of life (EUROHIS) (n=869) | | 3.8 (0.8) | 3.7 (0.7) | 3.8 (0.7) |
| Gender (n=882) | |  |  |  |
|  | Male | 53.8% | 56.8% | 30.8% |
|  | Female | 46.2% | 43.2% | 69.2% |
| Marital status (n=872) | |  |  |  |
|  | Single | 95.3% | 59.7% | 15.3% |
|  | Divorced | 1.2% | 32.6% | 33.1% |
|  | Widowed | 0.0% | 3.9% | 46.5% |
|  | Married/cohabiting | 3.5% | 3.9% | 5.1% |
| In a relationship (n=864) | |  |  |  |
|  | No | 57.0% | 76.7% | 87.2% |
|  | Yes | 43.0% | 23.3% | 12.8% |
| Education (n=876) | |  |  |  |
|  | Primary | 4.1% | 10.1% | 44.2% |
|  | Secondary | 66.3% | 36.3% | 23.3% |
|  | Tertiary | 29.7% | 53.6% | 32.5% |
| Employment status (n=870) | |  |  |  |
|  | Employed/studying | 91.7% | 71.3% | 1.0% |
|  | Unemployed | 8.3% | 23.0% | 3.5% |
|  | Retired/other | 0.0% | 5.7% | 95.5% |
| Region (NUTS2) (n=884) | |  |  |  |
|  | Helsinki-Uusimaa | 31.2% | 28.5% | 22.0% |
|  | South Finland | 24.9% | 24.1% | 28.0% |
|  | West Finland | 22.0% | 24.1% | 24.8% |
|  | East & North Finland | 22.0% | 23.3% | 25.2% |
| Urbanicity (n=870) | |  |  |  |
|  | City / town centre | 42.1% | 26.7% | 28.3% |
|  | City/town suburb | 50.3% | 54.7% | 43.6% |
|  | Population centre in a rural area | 7.6% | 10.4% | 20.4% |
|  | Sparsely populated rural area | 0.0% | 8.3% | 7.6% |
