# Supplementary material for: Profiles of subjective health among people living alone: a latent class analysis
Source: BMC Public Health. 2021 Jul 7;21:1335. doi: 10.1186/s12889-021-11396-2 (PMC8261976; doi:10.1186/s12889-021-11396-2)
Supplement: Supplementary file 2 — Additional file 2. Socio-demographic distributions and the 95% CIs of the latent groups. [file 12889_2021_11396_MOESM2_ESM.docx]

**Additional file 2** Socio-demographic distributions and the 95% CIs of the latent groups**.**

|  |  | Latent group | | | | | | | |
| --- | --- | --- | --- | --- | --- | --- | --- | --- | --- |
|  |  | Languishing (n=35) | | Managing (n=374) | | Healthy (n=266) | | Flourishing (n=274) | |
|  |  | % | 95% CI | % | 95% CI | % | 95% CI | % | 95% CI |
| Gender (n=882) | |  |  |  |  |  |  |  |  |
|  | Male | 71.6% | [55.6%; 87.5%] | 46.0% | [40.4%; 51.6%] | 49.4% | [43.4%; 55.5%] | 41.9% | [36.0%; 47.8%] |
|  | Female | 28.4% | [12.5%; 44.4%] | 54.0% | [48.4%; 59.6%] | 50.6% | [44.5%; 56.6%] | 58.1% | [52.2%; 64.0%] |
| Age (n=884) | |  |  |  |  |  |  |  |  |
|  | 18-29 years | 25.9% | [10.7%; 41.1%] | 13.6% | [9.7%; 17.4%] | 19.4% | [14.6%; 24.2%] | 25.3% | [20.1%; 30.4%] |
|  | 30-64 years | 52.7% | [35.3%; 70.0%] | 39.5% | [34.0%; 45.0%] | 46.5% | [40.5%; 52.6%] | 46.3% | [40.4%; 52.2%] |
|  | >65 years | 21.4% | [7.2%; 35.7%] | 46.9% | [41.3%; 52.5%] | 34.0% | [28.3%; 39.8%] | 28.4% | [23.1%; 33.8%] |
| Marital status (n=872) | | |  |  |  |  |  |  |  |
|  | Single | 73.9% | [58.6%; 89.2%] | 43.7% | [38.0%; 49.3%] | 54.5% | [48.5%; 60.6%] | 52.6% | [46.6%; 58.5%] |
|  | Divorced | 14.3% | [2.1%; 26.4%] | 27.4% | [22.3%; 32.5%] | 25.6% | [20.3%; 30.9%] | 28.4% | [23.0%; 33.8%] |
|  | Widowed | 11.9% | [0.6%; 23.1%] | 24.5% | [19.6%; 29.4%] | 15.3% | [10.9%; 19.6%] | 15.1% | [10.8%; 19.4%] |
|  | Married/cohabiting | 0.0% | [0.0%; 0.0%] | 4.4% | [2.0%; 6.7%] | 4.6% | [2.0%; 7.1%] | 3.9% | [1.6%; 6.2%] |
| In a relationship (n=864) | | |  |  |  |  |  |  |  |
|  | No | 3.5% | [0%*; 10.1%] | 19.1% | [14.5%; 23.6%] | 18.9% | [14.1%; 23.7%] | 35.4% | [29.7%; 41.1%] |
|  | Yes | 96.5% | [89.9%; 100%*] | 80.9% | [76.4%; 85.5%] | 81.1% | [76.3%; 85.9%] | 64.6% | [58.9%; 70.3%] |
| Education (n=876) | |  |  |  |  |  |  |  |  |
|  | Primary | 22.2% | [7.7%; 36.6%] | 32.1% | [26.8%; 37.4%] | 16.5% | [12.0%; 21.0%] | 13.5% | [9.5%; 17.6%] |
|  | Secondary | 60.0% | [42.9%; 77.0%] | 35.4% | [29.9%; 40.8%] | 40.4% | [34.4%; 46.3%] | 34.1% | [28.5%; 39.8%] |
|  | Tertiary | 17.8% | [4.5%; 31.2%] | 32.6% | [27.3%; 37.9%] | 43.1% | [37.1%; 49.1%] | 52.3% | [46.4%; 58.3%] |
| Employment status (n=870) | | |  |  |  |  |  |  |  |
|  | Employed/studying | 47.0% | [29.6%; 64.4%] | 33.8% | [28.4%; 39.1%] | 54.5% | [48.4%; 60.5%] | 64.2% | [58.5%; 70.0%] |
|  | Unemployed | 31.4% | [15.2%; 47.5%] | 20.9% | [16.3%; 25.5%] | 8.7% | [5.3%; 12.2%] | 6.4% | [3.5%; 9.4%] |
|  | Retired/other | 21.6% | [7.3%; 35.9%] | 45.3% | [39.7%; 51.0%] | 36.8% | [30.9%; 42.6%] | 29.4% | [23.9%; 34.8%] |
| Region (NUTS2) (n=884) | | |  |  |  |  |  |  |  |
|  | Helsinki-Uusimaa | 22.3% | [7.9%; 36.8%] | 23.7% | [18.9%; 28.4%] | 26.7% | [21.4%; 32.1%] | 30.5% | [25.1%; 36.0%] |
|  | South Finland | 33.1% | [16.7%; 49.5%] | 27.2% | [22.2%; 32.2%] | 27.0% | [21.6%; 32.4%] | 21.6% | [16.7%; 26.5%] |
|  | West Finland | 17.5% | [4.2%; 30.7%] | 27.0% | [22.0%; 32.0%] | 21.6% | [16.6%; 26.6%] | 23.6% | [18.5%; 28.6%] |
|  | East & North Finland | 27.1% | [11.6%; 42.6%] | 22.1% | [17.4%; 26.8%] | 24.7% | [19.5%; 29.9%] | 21.9% | [19.1%; 29.3%] |
| Urbanicity (n=870) | |  |  |  |  |  |  |  |  |
|  | City / town centre | 37.3% | [20.5%; 54.2%] | 31.1% | [25.8%; 36.4%] | 28.7% | [23.2%; 34.2%] | 30.0% | [24.5%; 35.5%] |
|  | City/town suburb | 43.7% | [26.4%; 60.9%] | 48.0% | [42.4%; 53.7%] | 52.0% | [46.0%; 58.1%] | 50.8% | [44.8%; 56.8%] |
|  | Population centre in a rural area | 16.9% | [3.8%; 29.9%] | 14.5% | [10.5%; 18.5%] | 14.2% | [9.9%; 18.4%] | 10.8% | [7.1%; 14.5%] |
|  | Sparsely populated rural area | 2.1% | [0%*; 7.1%] | 6.4% | [3.6%; 9.2%] | 5.1% | [2.4%; 7.7%] | 8.3% | [5.0%; 11.6%] |

* Constrained to 0% due to an estimate <0%

** Constrained to 100% due to an estimate >100%
