# Supplementary material for: Profiles of subjective health among people living alone: a latent class analysis
Source: BMC Public Health. 2021 Jul 7;21:1335. doi: 10.1186/s12889-021-11396-2 (PMC8261976; doi:10.1186/s12889-021-11396-2)
Supplement: Supplementary file 3 — Additional file 3. Covariate-only model explaining quality of life (EUROHIS-QOL8). [file 12889_2021_11396_MOESM3_ESM.docx]

**Additional file 3** Covariate-only model explaining quality of life (EUROHIS-QOL8), n=809, R^2^=.15. Estimates **in bold**: p<.05. CI = compatibility interval.

|  |  | b | 95% CI | p |
| --- | --- | --- | --- | --- |
| Gender (male vs female) | | -0.08 | [-0.19; 0.04] | .191 |
| Age group (ref. <30 years) | |  |  |  |
|  | 30-64 years | -0.06 | [-0.27; 0.15] | .587 |
|  | >65 years | 0.06 | [-0.22; 0.34] | .686 |
| Marital status (ref. single) | |  |  |  |
|  | Married/cohabiting | -0.09 | [-0.34; 0.16] | .478 |
|  | Divorced/separated | 0.02 | [-0.11; 0.15] | .740 |
|  | Widowed | **0.16** | [0.01; 0.31] | .034 |
| Employment status (ref. employed/studying) | | | |  |
|  | Unemployed | **-0.58** | [-0.79; -0.36] | <.001 |
|  | Retired/other | -0.11 | [-0.32; 0.1] | .290 |
| In a relationship (vs not) | | **0.23** | [0.07; 0.4] | .005 |
| Education level (ref. comprehensive) | |  |  |  |
|  | Secondary | 0.02 | [-0.12; 0.16] | .749 |
|  | Higher | **0.24** | [0.11; 0.37] | <.001 |
| Region (NUTS2; ref. South Finland) | |  |  |  |
|  | Helsinki-Uusimaa | 0.07 | [-0.06; 0.21] | .276 |
|  | West Finland | -0.07 | [-0.24; 0.1] | .418 |
|  | East & North Finland | 0.01 | [-0.19; 0.2] | .931 |
| Urbanicity (ref. city/town centre) | |  |  |  |
|  | City/town suburb | 0.1 | [-0.06; 0.25] | .211 |
|  | Population centre in a rural area | 0.07 | [-0.09; 0.22] | .403 |
|  | Sparsely populated rural area | 0.14 | [-0.03; 0.3] | .101 |
