# Supplementary material for: Profiles of subjective health among people living alone: a latent class analysis
Source: BMC Public Health. 2021 Jul 7;21:1335. doi: 10.1186/s12889-021-11396-2 (PMC8261976; doi:10.1186/s12889-021-11396-2)
Supplement: Supplementary file 4 — Additional file 4. BCH-corrected LCA model with covariates and quality of life (EUROHIS-QOL8) as distal outcome. [file 12889_2021_11396_MOESM4_ESM.docx]

**Additional file 4** BCH-corrected LCA model with covariates and quality of life (EUROHIS-QOL8) as distal outcome, n=824. Estimates **in bold**: p<.05. CI = compatibility interval.

|  |  | BCH-corrected model | | |
| --- | --- | --- | --- | --- |
|  |  | b | 95% CI | p |
| Group intercepts | Languishing | **2.58** | [2.13; 3.03] | 0.00 |
|  | Managing | **3.18** | [2.98; 3.37] | 0.00 |
|  | Healthy | **3.81** | [3.63; 3.99] | 0.00 |
|  | Flourishing | **4.41** | [4.22; 4.61] | 0.00 |
| Gender (male vs female) | Male | 0.02 | [-0.08; 0.12] | 0.73 |
| Age group (ref. "<30") | | |  |  |
|  | 30-64 | 0.00 | [-0.17; 0.17] | 0.98 |
|  | >65 | 0.16 | [0; 0.32] | 0.19 |
| Marital/relationship status (ref. single)* | | |  |  |
|  | Married/cohabiting/in a relationship | -0.02 | [-0.15; 0.12] | 0.78 |
|  | Divorced/separated | -0.04 | [-0.15; 0.08] | 0.55 |
|  | Widowed | 0.11 | [-0.02; 0.25] | 0.09 |
| Employment status (ref. employed/studying) | | | |  |
|  | Unemployed | **-0.23** | [-0.42; -0.05] | 0.01 |
|  | Retired/other | -0.04 | [-0.21; 0.15] | 0.71 |
| Education level (ref. primary) | | | |  |
|  | Secondary | -0.03 | [-0.16; 0.1] | 0.67 |
|  | Tertiary | 0.00 | [-0.12; 0.12] | 0.95 |
| Urbanicity (ref. city/town centre) | |  |  |  |
|  | City/town suburb | 0.03 | [-0.07; 0.14] | 0.54 |
|  | Rural** | **-0.12** | [-0.23; 0] | 0.05 |

*All respondents ‘in a relationship’ included in the first category, and others categorised based on their marital status

**Combined population centre in a rural area / sparsely populated rural area
