# Supplementary material for: Profiles of subjective health among people living alone: a latent class analysis
Source: BMC Public Health. 2021 Jul 7;21:1335. doi: 10.1186/s12889-021-11396-2 (PMC8261976; doi:10.1186/s12889-021-11396-2)

**Additional file 5** Socio-demographic distributions of the latent groups, weighed by classification probabilities using the BCH method. Due low frequencies in group Languishing that caused issues in estimation, marital and relationship status were merged into one variable.


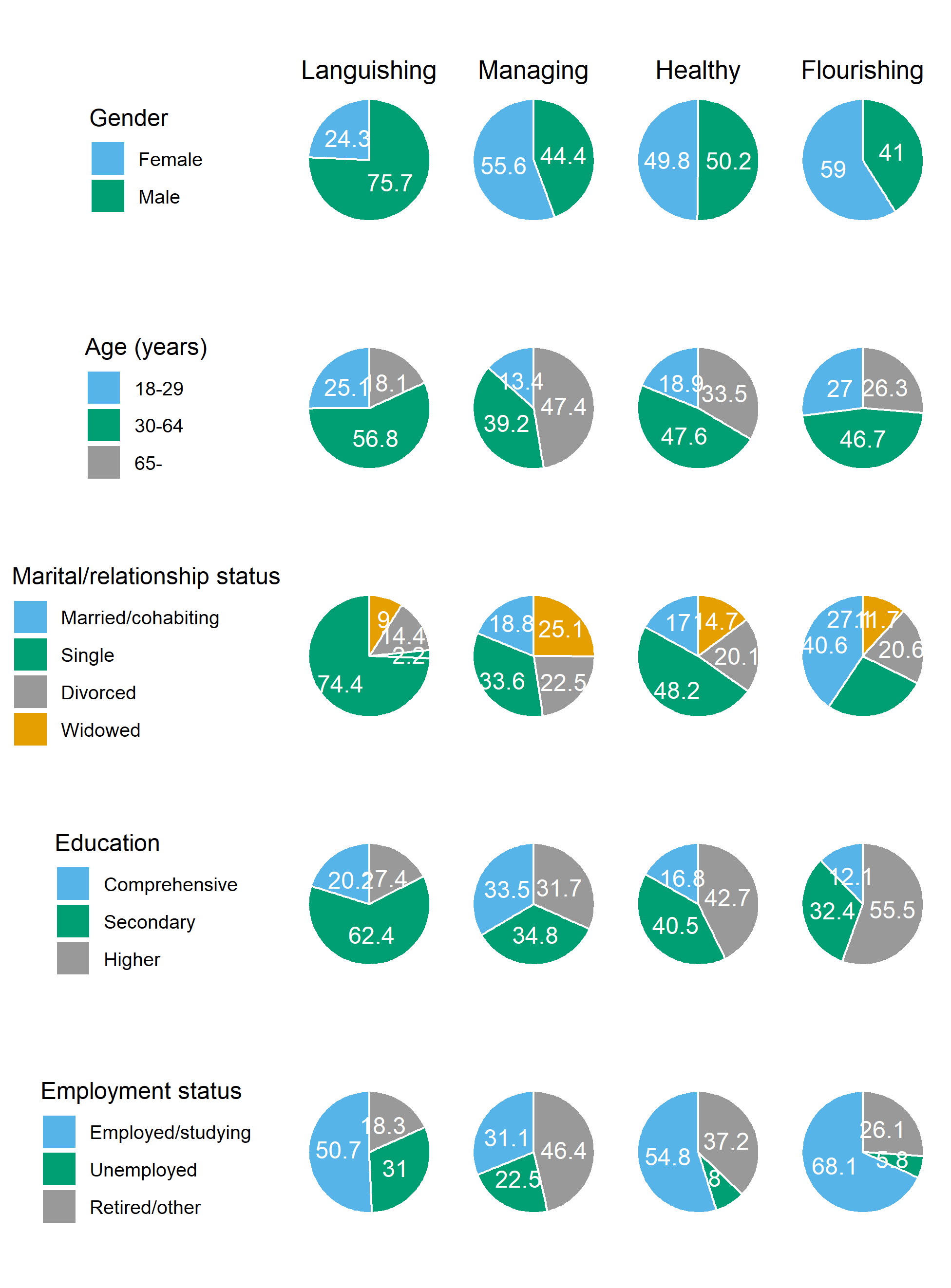

Supplement: Supplementary file 5 — Additional file 5. Socio-demographic distributions of the latent groups, weighed by classification probabilities using the BCH method. [file 12889_2021_11396_MOESM5_ESM.docx]
